# Gendered Disease Iconography through the Lens of COVID-19 in Hong Kong

**DOI:** 10.1177/1097184X211001415

**Published:** 2021-03-11

**Authors:** Harry Yi-Jui Wu

**Affiliations:** 1 University of Hong Kong, Pok Fu Lam, Hong Kong

**Keywords:** toxic masculinity, disease iconography, Hong Kong, politics of care, COVID-19

## Abstract

Amidst the rampancy of COVID-19, the news media and academics have emphasized men’s gendered vulnerability. Many examine the risks individuals or communities are exposed to, implying the need to reconsider gendered stereotypes that victimize people at work and at home. In this short commentary, rather than interrogating the risk-related agenda, I reflect on genders as imagined sources of disease by studying strategies adopted by administrators in health communication for the purpose of disease control.

Using AIDS and syphilis as examples, Sander Gilman (Gilman 1988) notes that the construction of various boundaries of disease, of images of the patient as the vessel and transmitter, depends on our moral sense and consequent desire to insulate those we define as ill. COVID-19 exposed people’s speculations about the reservoirs of pathogens. In a post-conflict society, Hong Kong is an ideal looking glass, magnifying the process through which symbols of disease are portrayed. On the one hand, this short essay aims to examine how gendered sources of COVID-19 manifest in a society within a specific geopolitical context. On the other hand, through the new iconography of disease, I identify the challenges of modernity “Asia’s world city” has been experiencing. I explore these questions through an examination of specific aspects of Hong Kong’s response to COVID-19: gendered Sinophobia toward police officers’ wives; reactions to retired women who sought out younger men as dance partners during COVID-19; failure to recognize and protect foreign domestic workers as a vulnerable group during the pandemic; government actions that draw on “toxic masculine” traits and values; the city’s response to queer communities’ involvement in COVID-19 outbreaks; and the government’s deliberate de-emphasis on gender issues as compared with China. Each of these examples encapsulates how both gendered and cross-border conceptions of disease iconography were geopolitically formed regarding the recent tension between Hong Kong and China.

In early 2020, the naming of the newly discovered coronavirus disease unleashed immense global debate. Many discussions focused on Sinophobia and its adverse effects in public health practice. Fearing a repetition of its 2003 experience of SARS, Hong Kong became a hotbed of such disputes (Chung and Li 2020). General concerns about the care burden and other gender-based family issues during lockdowns, however, did not surface as much as elsewhere (Catarina et al. 2020; Dlamini 2020). Public health measures in Hong Kong instead focused on transportation control between the special administration region and mainland China. Due to stricter measures taken at the border to prevent carriers of pathogens from entering, a lockdown did not occur. Alongside the border controls, preceding the disease outbreak, hatred in the city toward foreign communities, predominately Chinese people, was already prevalent.

Public imaginations on gender and disease were already entwined with nationality implications in Hong Kong long before the COVID-19 outbreak, revealing its tension with China. During the year-long pro-democracy protest in 2019, in addition to the pro-establishment political figures, police became the primary targets of aggression, both in street protests and online (Chang 2020). Women became the unfortunate victims of such aggression displaced from Hong Kong people’s revulsion against police violence. For example, during the year-long protest, foul language was used to humiliate the wives of those police officers who are men; the remarks expressed during the anti-Extradition Law Amendment Bill protest, for instance, eventually attracted minimal attention. Following these sentiments, wives of police officers (*keng-so* in the Cantonese pronunciation) became an unfortunate target during the COVID-19 pandemic, situated both as victims and sources of the disease—a portrayal no different from the iconography portraying them in early modern Europe. At the end of January, for example, when the first suspected infection of the wife of a riot police member appeared in the news, messages expressing “Congratulations” went viral on the Internet. For example, appearing in the online forum HA (Hospital Authority) Secrets on January 26, 2020, a post numbered #9693 congratulated the officer’s wife: “Look at how cheap you are. Your husband have [sic] been beating up people and receiving HKD 900 million bonus. While you might die from COVID for nothing, your husband can marry another wife from the Mainland or rape young girls at the police station. I congratulate you for that.” When the lockdown in Wuhan was lifted, after another police officer’s wife expressed her desire to appreciate the cherry blossoms therein, similar derision was conveyed on the Internet. In these posts, police officer’s wives regrettably became a metaphor of a disease reservoir, discursively relied on as props to address the actual targets of this hateful rhetoric: policemen.

In another incident of how gender connects to group infection, rich and corrupt housewives became targets of collective venting. In November of 2020, Hong Kong reported the highest number of new infections in about three months. Among the cases, the majority were linked to a cluster of dance club members (Wang 2020). Ultimately, more than 700 people were directly involved in the outbreak. Several leaked video clips from an associated club showed members of the dance halls, mostly retired women, enjoying the company offered by the much younger men instructors. These instructors were from lower social and economic statuses, spending nights in Hong Kong to earn cash from wealthy women in the city. An HKTV program revealed their poor living conditions in crowded subdivided flats, exposing them to greater risk of infection (Lee 2020). Similarly, with pity cast on the financially deprived instructors, these rich women again became objects of ridicule. For example, an elderly woman licking the nipple of her much younger dance partner soon became an animated sticker on WhatsApp. In addition to such discontent expressed about rich people with vested interests, netizens exerted their rage toward China, where the pathogen perceivably originated.

Another unique phenomenon in the city was the impact of the pandemic on the main caregivers in the cosmopolitan city: foreign domestic workers. According to recent statistics, by the end of 2019, there were more than 399,320 women domestic workers in Hong Kong, predominantly from the Philippines and Indonesia (Immigration Department 2020). While most discourse thus far has focused on the burden suffered by women who had to work from home while taking care of children, elderly relatives, and other home affairs, the emerging domestic workers whose welfare was neglected revealed a narrow imagination regarding care in a supposedly globalized city. In Hong Kong, foreign domestic workers are more than caregivers. They are often required to partner with women employers in the home. As burden sharers, they have little bargaining power while negotiating work with housewives who do not necessarily behave as feminist allies. The employees are institutionally and socially discriminated against (Hui and Liang 2020), and usually suffer from verbal or other types of aggression even from the women employing them, who might feel their position as a spouse or a mother in the family is undermined or threatened (Lai and Fong 2020). Early in the pandemic there was little regard for protecting domestic workers’ health. Such loopholes in health governing did not emerge until later, however, when employers were asked to pay the cost of treatment, necessary quarantine, or hospital admission if domestic workers were infected. Although they are an indispensable component of the transnational in Hong Kong, their wellbeing had not been attended to until the potential risk they were exposed to finally emerged through the pandemic.

Men’s behaviors that are stoic, emotionless, domestic, and out-of-touch in the context of caregiving are sometimes popularly referred to as “toxic masculinity”. These behaviors often result in ineffectiveness in solving problems. Such characteristics were revealed by the stubborn style of how various Hong Kong authorities dealt with COVID-19. For example, in July 2020, when Hong Kong saw a surge in the number of infections, one of the policies banned people from having lunch in indoor spaces, leaving significant numbers of construction workers eating in heavy rain. In this case, it was not the tough and risk-taking behaviors of the predominantly male workers that were putting them at risk (e.g., Betron et al. 2020; Mellström 2020; Palmer and Peterson 2020). Rather, here, indifferent and unreflective authority gave rise to men’s increased vulnerability. The government received extensive criticism, resulting in halting the order after one day. Some officials claimed they were unaware so many people could not work from home and had to dine out. In another example, when the authorities faced the massive gatherings appearing on weekends, mostly consisting of foreign domestic workers, they were unable to effectively force the crowds (which bore a high risk of infection) to follow the rule of social distancing for diverse reasons. First, the aggression of law enforcement officers was normally expressed toward local citizens. Police were frequently deployed to target “unlawful assemblies” of a smaller scale, either in the street or indoors (Frazier 2020). Second, authorities possibly realized that the crowds were also essential to the city’s economic sustainability.

Despite the toxic masculine style of governance during the pandemic, several gender issues in Hong Kong have been deliberately played down, revealing the necessary forbearance of plurality as a cosmopolitan city compared with other Asian cities. One the one hand, the tolerance shows an indifferent attitude toward unconventional gender identities in a conservative and patriarchic society. On the other hand, such lenience was essential to the maintenance of both middle-class family doing and to the consumer culture essential to the city’s prosperity (Yue and Leung 2015), and included what may seem to be a surprising lack of sensationalism in reaction to outbreaks among queer residents. For example, the homophobic panic that surfaced after a coronavirus cluster appeared in Itaewon in Seoul, when government contact tracing efforts raised fears of public outing – did not occur in Hong Kong. An early 2020 infection incident at a gay bar in Hong Kong likewise did not cause an uproar, perhaps partially because it did not result in a large outbreak. At the daily press conference of Hong Kong’s Department of Health, moreover, the fact that the venue was a gay bar was deemphasized.

The case of COVID-19 control measures in Hong Kong revealed how its unique geopolitical context caused a reaction that was dramatically different from the general concerns about the impact of COVID-19 on gender issues in most English writing. Unlike gender-making exercises led by the party-state in Beijing, the special administrative region still preserved its pragmatic character. In February 2020, during the most intense days of the initial viral outbreak in China, women medical workers there shaved their hair for hygienic and convenience purposes. The “handsome beautiful worriers” campaign was portrayed in state media but later received criticism from Chinese feminists (Stevenson 2020). In Hong Kong, such curative political performances were less obvious. With its benefits have yet to be evaluated, making light of gender before and during COVID-19 was a measure implicitly taken to preserve the city’s international status and to attract worldwide talent to bolster the region’s prosperity.

In Hong Kong, the creation of disease iconography not only reflects the recent political tension between the special administrative region and China but also signifies the society’s conservative and macho attitude toward women. Echoing Gilman’s (1988) argument, icons of disease first appeared to exist independent of the reality of any given disease. The “free-floating” iconography attached to COVID-19 in the city, however, shows how the gendered representation of disease reservoirs operates in the context in which such imagination functions. As an international cosmopolitan city, although gender stereotypes have been downplayed by the administrators regarding disease control, certain gender identities or temperaments are still divulged and reinforced regarding social attitudes toward the matter of “care” and public health measures. These identities are often carried forward with class, race, nationalities, and other social categories, suggesting a restricted conventional understanding of gender and health in existing scholarly accounts. The iconography of COVID-19 in Hong Kong alerts us to how gendered subjects resist stereotypical assumptions and how they continue to expand and transform within a context structured by a tapestry of patriarchal Chinese identity, immigration, capital flow, and political tensions being continuously (un)woven.
